# Clinical Lectures on Diseases of the Urinary Organs

**Published:** 1875-08

**Authors:** 


*Clinical Lectures on Diseases of the Urinary Organs.* Delivered at University College Hospital. By Sir Henry Thompson. Philadelphia: Henry C. Lea, 1875. T. Butler & Son, pp 195.

These lectures which have reached a third edition in England and a second in this country, comprise in a small space a vast amount of instruction.

The introductory chapter on Diagnosis is excellent, and should be read by every student of the diseases of the Urinary Organs. The advice given is terse and to the point, and if more frequently followed would prevent some of the unfortunate errors which occasionally occur in this line of practice.

The lectures are fourteen in number being an addition of two over the former edition. They consider stricture, the treatment of stricture, hypertrophy of the prostate, retention of urine, extravasation of urine and fistulæ, stone in the bladder, lithotrity, lithotomy, the early history of calculous disease, cystitis and prostatitis, diseases of the bladder, and hæmaturia and renal calulus. In the treatment of stricture dilatation is advised always in every case, where this will not succeed internal urethrotomy is advised, the author giving preference to the instrument of Civiale. He does not speak very highly of the method of overdistension or of the instrument invented by himself for this purpose. In relation to the early history of calculous disease he says: "A very simple rule, indeed, too simple, I think, is often adopted. When the urine has persistently and habitually thrown down acid deposits, and the patient has generally been prescribed alkalies; if on the contrary he has alkaline deposits, he has been treated with acids. * * * * * You really have the same condition as that of the fabled ostrich, which is said to put its head in the bush when pursued by hunters, and, no longer seeing them, believes itself secure. Just such is the security of the patient with uric acid who trusts solely to alkalies or Vichy water. His surplus deposits have become imperceptible to *his* vision; nothing more." The treatment advised by the author is such as will stimulate the excretory action by the primæ viæ without depressing the vital power. For this purpose the mineral waters containing the sulphates of soda and of magnesia in considerable proportion are recommended, and he finds that those of Friedrichshalle are the most desirable.

Were time and space to allow we should be pleased to speak of this work to an greater extent, but we can only add that the lectures are admirably adapted to the wants of those in search of information in this department of medicine.

